# Repeat hepatectomy with systemic chemotherapy might improve survival of recurrent liver metastasis from colorectal cancer—a retrospective observational study

**DOI:** 10.1186/s12957-019-1575-y

**Published:** 2019-02-15

**Authors:** Hiroshi Matsuoka, Zenichi Morise, Chihiro Tanaka, Takahiro Hayashi, Yoshiaki Ikeda, Koutarou Maeda, Koji Masumori, Yoshikazu Koide, Hidetoshi Katsuno, Yoshinao Tanahashi, Sanae Nakajima, Tsunekazu Hanai, Yutaro Kato, Atsushi Sugioka, Ichiro Uyama

**Affiliations:** 1 Department of Surgery Fujita Health University, 1-98 Dengakugakubo Kutsukake-cho, Toyoake City, Aichi 470-1192 Japan; 2 College of Pharmacy, Kinjo Gakuin University, 2-1723 Oomori Moriyama, Nagoya City, Aichi 463-8521 Japan; 3 Fujita Health University Hospital International Medical Center, 1-98 Dengakugakubo Kutsukake-cho, Toyoake city, Aichi 470-1192 Japan

**Keywords:** Liver metastases, Repeat hepatectomy, Colorectal cancer, Systemic chemotherapy

## Abstract

**Background:**

Although hepatectomy for metastatic colorectal cancer (mCRC) prolongs survival in up to 40% of people, recurrence rates approach 70%. We used a multidisciplinary approach to treat recurrent liver metastases, including chemotherapy, surgery, and palliative care. On the other hand, development of chemotherapeutic agents is remarkable and improves long-term survival. However, whether chemotherapy and repeat hepatectomy combination therapy improve survival or not is still unclear. The aim of this study was to analyze the outcomes of repeat hepatectomy with systemic chemotherapy for mCRC.

**Methods:**

Following Institutional Review Board approval, we reviewed the records of all patients who underwent hepatectomy for mCRC between 1974 and 2015 at Fujita Health University Hospital. We used the Kaplan–Meier method to estimate overall survival from the first and last hepatectomy in multi hepatectomy cases after 2005 and compared outcomes between groups using the log-rank test.

**Results:**

A total of 426 liver resections were performed for mCRC; of these, 236 cases were performed after 2005 (late group). In 118 (50%) cases, the site of recurrence was the liver, 59 (50%) underwent repeat hepatectomy, and 14 cases had ≥ 2 repeat hepatectomies.

Overall survival (OS) before and after 2005 was 42.2 and 64.1 months, respectively, with the late group having better OS compared to the early (1974–2004) group. OS for single hepatectomy cases was 83.2 months, for two hepatectomies was 42.9 months, and for three hepatectomies was 35.3 months. In total, 59 patients did not undergo surgery after recurrence with an OS of 28.7 months. Mortality of the second and third repeat hepatectomy was 1.7% and 15.3%, respectively.

**Conclusion:**

Repeat hepatectomy with systemic chemotherapy for mCRC is feasible and might achieve improved survival in carefully selected patients.

## Background

Liver metastasis is the strongest determinant for prognosis in patients with advanced and recurrent colorectal cancer (CRC). Synchronous liver metastasis occurs in approximately 15% of patients with CRC at initial treatment, and liver metastasis occurs in approximately 50% of patients during follow-up for CRC [1, 2]. Therefore, improving treatment results for liver metastasis improves outcomes of CRC patients. Liver metastasis of CRC is treated by surgical resection if radical resection is feasible; however, remnant liver recurrence has been reported in approximately 60–70% of patients after initial hepatectomy [3]. We reported previously that the aggressive implementation of repeat hepatectomy for remnant liver recurrence prolongs survival [4]. Morise et al. reported 5-year survival of 188 hepatectomies for metastatic liver cancer from CRC between 1974 and 2004 and found that repeat hepatectomy for remnant liver metastases improves OS. In this report, the single hepatectomy group’s 5-year survival from first hepatectomy is 41.4%, and the three times hepatectomy group’s 5-year survival from first hepatectomy is 53.9%. At this time, the only systemic chemotherapeutic agent is 5-fluorouracil (5-FU). At the time of remnant liver recurrence, the patient had been received 5-FU and refractory. Actually, new chemotherapeutic drugs like Oxaliplatin (L-OHP), irinotecan hydrochloride (CPT-11), and molecular target drugs was not performed in this study period. Conversely, although various attempts have been made to improve treatment results, it appears that advances in chemotherapy have been the most influential. The advent of L-OHP and CPT-11 has generated a dramatic change in drug therapy for CRC, for which 5-FU had been the only mainstay drug. Eventually, the molecular-targeted drugs bevacizumab, cetuximab, and panitumumab became available, and its combination with previous cytotoxic drugs enabled patient survival of ≥ 30 months for unresectable advanced/recurrent CRC [5]. The significance of repeat hepatectomy following standard treatment with these new drugs cannot be compared with 5-FU since it was the only effective drug at the time; however, research on this issue has rarely been reported. The development of systemic chemotherapy for advanced CRC introduces a conversion strategy; this strategy is to convert unresectable distant metastatic lesion into resectable by a chemotherapeutic effect. However, it is unclear whether these conversion cases improve OS. In this study, we compared data obtained before and after 2005 in terms of survival time, when FOLFOX (bolus and continuous 5-FU + leucovorin + L-OHP) therapy was introduced in Japan following the approval of L-OHP. In addition, we investigated the significance of additional chemotherapy with repeated hepatectomy which was adapted since 2005.

## Materials and methods

**Fig. 1:**
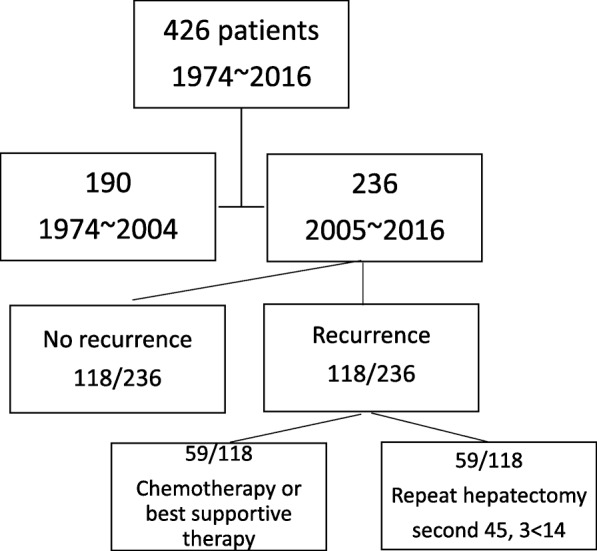
Flow chart of patients and procedure

In our hospital, hepatectomy was performed in 426 patients with liver metastasis from CRC from April 1974 to December 2016. Of these 426 patients, 190 underwent hepatectomy before December 2004 (early-period hepatectomy group), and 236 underwent hepatectomy during or after 2005 (late-period hepatectomy group). Remnant liver recurrence was seen in 118 (50%) patients in the later group. Among those who underwent repeat hepatectomy during or after 2005, 59 and 14 patients had 2 and ≥ 3 hepatectomies, respectively. Conversely, 59 patients with recurrence did not undergo surgery (Fig. 1). Table 1 shows the clinical characteristics of patients in the late (2005~2016) hepatectomy group. Overall survival (OS) of patients in the early and later period was determined based on data from the medical record. OS from the first and last hepatectomy was determined in the late multiple hepatectomy group. We used Microsoft Excel statistics software to perform statistical analysis. The Kaplan-Meier method was used to calculate survival time, and log-rank tests were used to compare the patient groups. We evaluated only survival data because the records of the previous period group had been discarded. Safety data, including the operating method, operation time, blood loss, complications, and duration of hospital stay after the operation were extracted for the late multiple hepatectomy group.

**Table 1:**
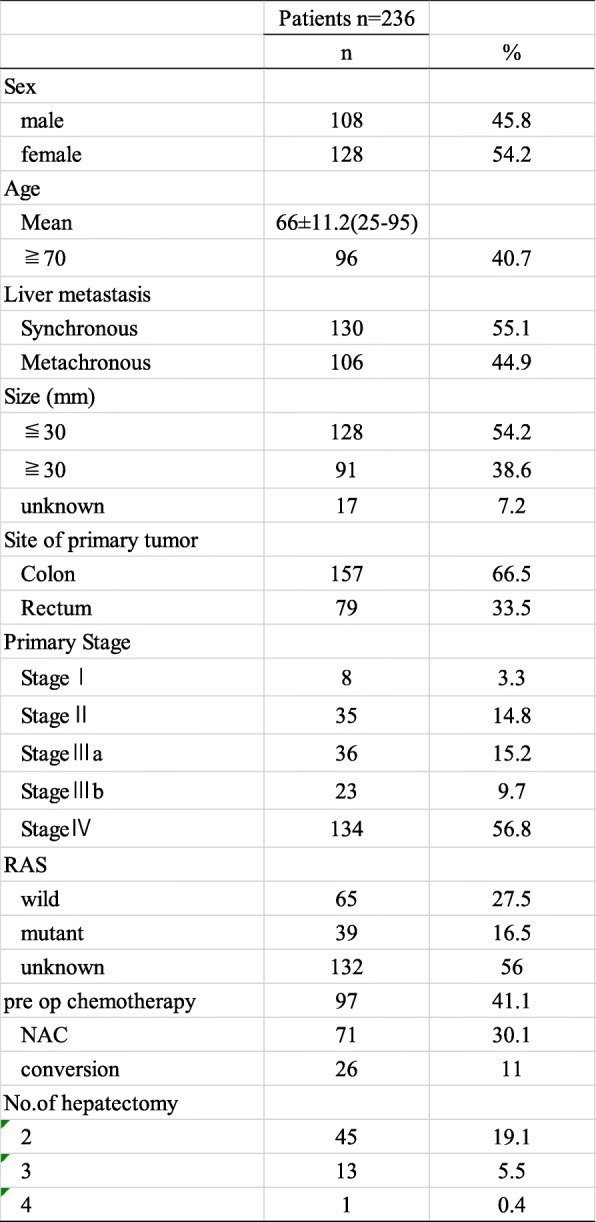
Patients characteristics of hepatectomy cases from mCRC after 2005

## Results

### Survival rate by period

**Fig. 2:**
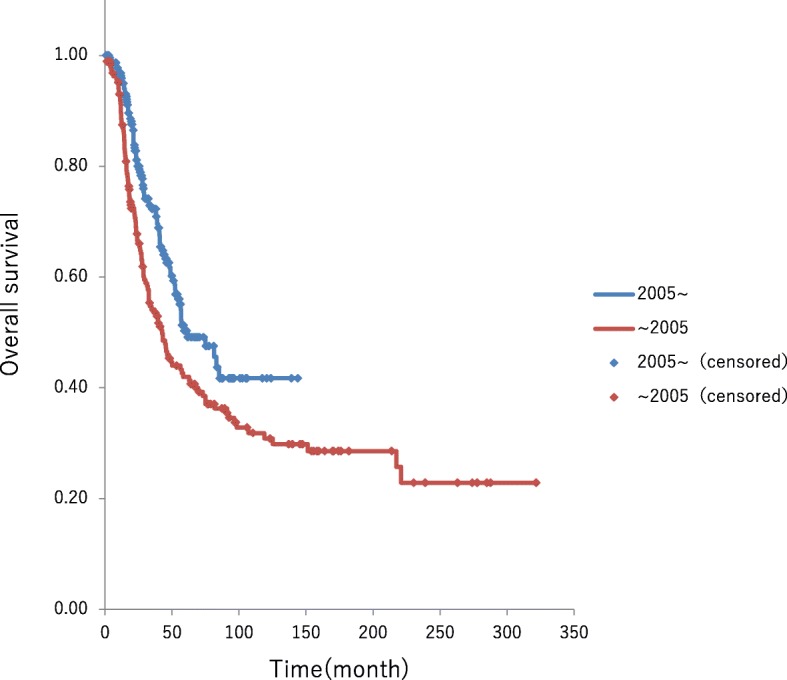
The median OS was 42.2 months in patients who underwent hepatectomy before 2004 and 61.1 months in who underwent hepatectomy during or after 2005, with a statistically significant survival time in the later group (*P* = 0.0064)

In patients who underwent hepatectomy by 2004, the median OS was 42.2 months and 61.1 months in those who underwent hepatectomy during or after 2005, with a statistically significant survival time in the later group (*P* = 0.0064) (Fig. 2). The following analyses used data for 236 cases during and after 2005:

### Survival time by Japanese criteria for complement factor H

**Fig. 3:**
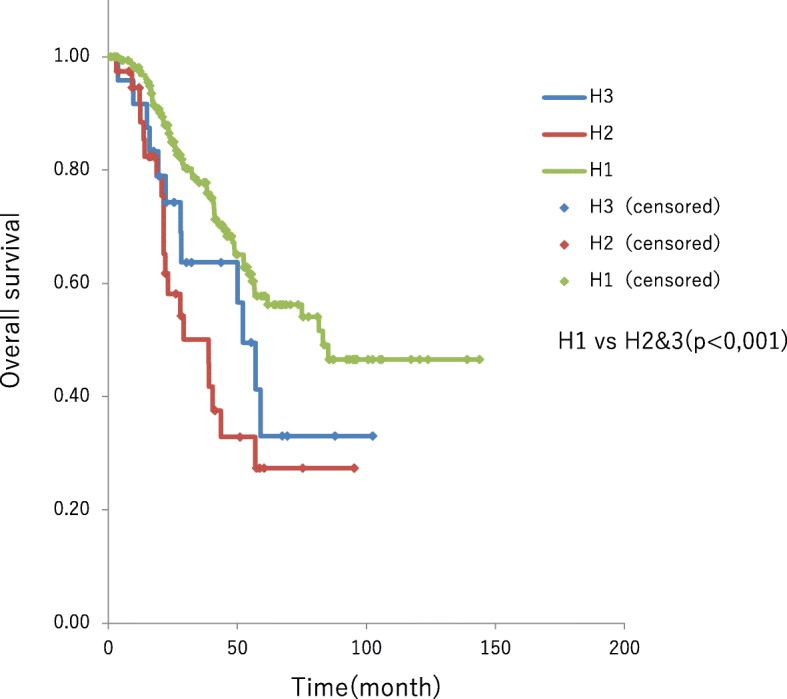
The median survival time was 83.2 months in patients with factor H1, 38.8 months in those with factor H2, and 52.3 months in those with factor H3. Patients with factor H1 had significantly better outcomes compared to those with factors H2 or H3 (*P* < 0.001)

Based on the *Japanese Classification of Colorectal Carcinoma 8th Edition* [6], factor H1 (< 4 pieces and maximum dimension < 5 cm) was found in 171 patients, factor H2 (excluding H1 and H3) in 41, and factor H3 (> 5 pieces and maximum dimension > 5 cm) in 24. The median survival time was 83.2 months in patients with factor H1, 38.8 months in those with factor H2, and 52.3 months in those with factor H3. Patients with factor H1 had significantly better outcomes compared to those with factors H2 or H3 (*P* < 0.001) (Fig. 3). Overall, 23 of the 24 patients with factor H3 had undergone preoperative chemotherapy. Overall, 19 patients with factor H3 had remnant liver recurrence, 9 of whom underwent repeat hepatectomy; hepatectomy was performed three times in 5 patients and four times in 1 patient. Survival time after the initial hepatectomy exceeded 30 months in 2 patients.

### Repeat hepatectomy

**Fig. 4:**
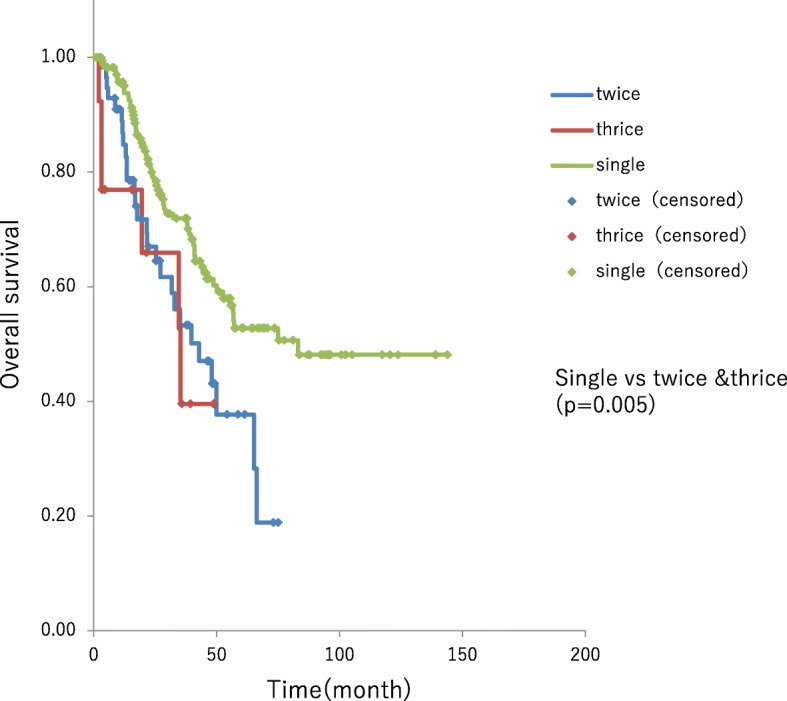
5-year survival rate of 78% in single hepatectomy cases and multiple hepatectomies. The median survival time after the last hepatectomy was 42.9 months and 35.3 months in patients undergoing second and third hepatectomy, respectively. Thus, the survival time was significantly longer in patients who underwent single hepatectomy (*P* = 0.005). However, there was no significant difference in patient outcomes in those undergoing hepatectomy two versus three times (*P* = 0.48)

Median survival time in patients who underwent single hepatectomy during or after 2005 was 83.2 months. Among these patients, those who were recurrence-free after single hepatectomy had a 5-year survival rate of 78% without reaching the median OS time. Conversely, among those who underwent multiple hepatectomies, the median survival time after the last hepatectomy was 42.9 months and 35.3 months in patients undergoing second and third hepatectomy, respectively. Thus, the survival time was significantly longer in patients who underwent single hepatectomy (median OS 83.2 month, 5-year survival 52.3%) (*P* = 0.005). However, there was no significant difference in patient outcomes in those undergoing hepatectomy two versus three times (*P* = 0.48) (Fig. 4).

**Fig. 5:**
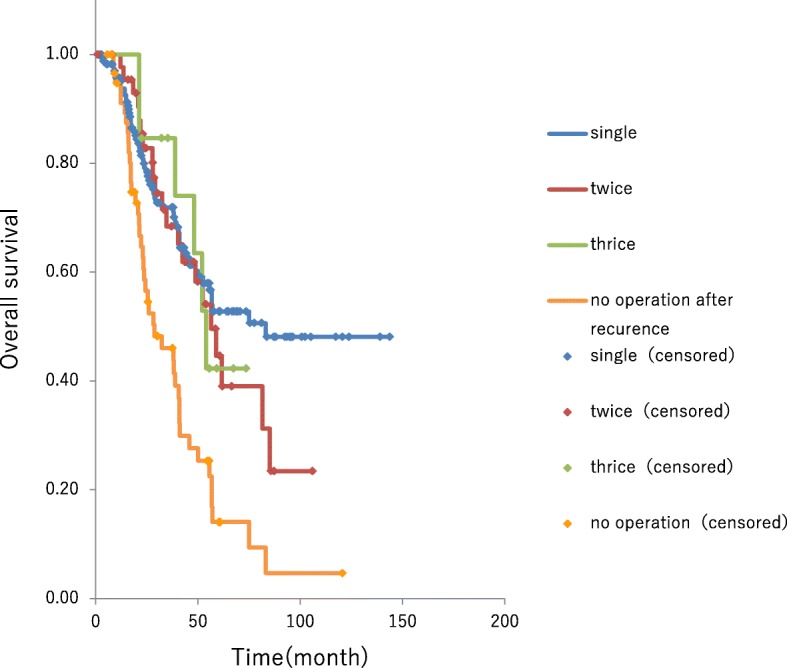
OS from initial hepatectomy was 56.5 months and 54.0 months in those undergoing two and the hepatectomies, respectively, neither showing significant differences in survival time compared to those undergoing single hepatectomy cases. The 59 patients who received palliative systemic chemotherapy due to an unresectable recurrent lesion had a median OS of 28.7 months, which was significantly shorter than was observed in the resection cases (*P* < 0.001)

Among the patients who underwent repeat hepatectomy, the survival time after the initial hepatectomy was 56.5 months and 54.0 months in those undergoing two and three hepatectomies, respectively, neither showing significant differences in survival time compared to those undergoing single hepatectomy. Conversely, among the 59 patients who received palliative systemic chemotherapy due to an unresectable recurrent lesion, the median OS was 28.7 months, which was significantly shorter than was observed in the resection cases (*P* < 0.001) (Fig. 5).

### Systemic chemotherapy

Two, three, and four regimens of systemic chemotherapy were administered to 30, 17, and 8 patients with repeat hepatectomy, respectively.

### Safety

The median hospital stay was 36.8 days after the initial hepatectomy, 42.8 days after a second hepatectomy, and 33.4 days after a third hepatectomy, and the differences were not significant. In patients who underwent repeat hepatectomy, 16 (28%) and 7 (53%) patients undergoing hepatectomy two and three times, respectively, had Clavien–Dindo grade ≥ 3 complications. Death occurred in one (1.7%) patient who had two hepatectomies and in two (15.3%) patients who had three hepatectomies. The cause of death in the two patients undergoing three hepatectomies was liver failure due to underlying chronic hepatitis (one patient) and excessive stress from massive hepatectomy (the other patient) with an operation time of 1032 min and surgical blood loss of 3116 mL (Table 2).

**Table 2:**
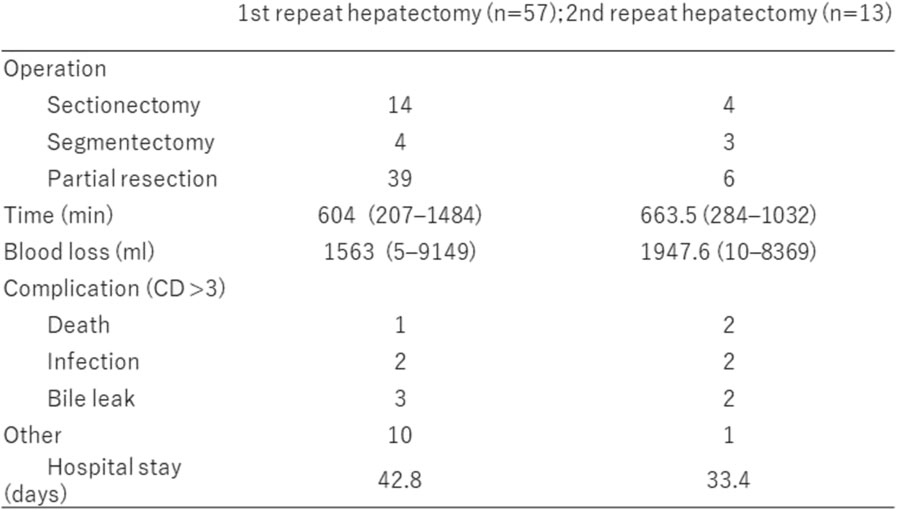
Perioperative date for repeat hepatectomy

## Discussion

Based on our experience, the survival time of patients with liver metastasis has increased with the changing times, and the approval of the new drugs after 2005 appears to have influenced this trend. We evaluated the impact on the survival of repeat hepatectomy in the age of advanced systemic chemotherapy. Does systemic chemotherapy alone, repeat hepatectomy, or a multidisciplinary approach improve the prognosis of liver metastases? There are many factors relevant to long-term survival of recurrent liver metastases, improvement of diagnostic imaging like multislice CT, PET, enhanced MRI; surgical instruments; surgeons’ learning curve; perioperative management; anesthesia; and supportive care. Although only a randomized controlled study can answer that question, such a study is prohibitive due to cost, time, and ethical concerns. Especially indication of surgical procedure is important, our principle indication does not change during all the period studied. We investigated the significance of repeat hepatectomy with systemic chemotherapy on outcomes of patients who have undergone standard treatment since 2005.

After the development of FOLFOX and FOLFIRI (5-FU + CPT-11 + leucovorin), the basic current standard treatment regimens since 2005, remnant liver recurrence occurred in 118 (50%) of 236 patients following the initial hepatectomy. Although this result was not noticeably different from that of the previous period, 59 (50%) of the 118 patients underwent repeat hepatectomy. OS time after the last hepatectomy decreased as the number of hepatectomies increased. However, patients who underwent repeat hepatectomy experienced survival time after the initial hepatectomy comparable to that in patients who underwent single hepatectomy. However, in a group of patients who were treated before the new drugs were approved, the survival time increased as the number of hepatectomies increased. Moreover, conversion cases (i.e., cases in which surgical resection was possible after chemotherapy) might have been included in this study. Preoperative chemotherapy was administered to 23 (96%) of the 24 patients with factor H3. Overall, 12 (50%) of the 24 patients were retrospectively regarded as conversion cases and were previously considered not favorable for surgery. Thus, the survival-prolonging effect of surgery alone appears to have diminished accordingly. Conversely, patients with remnant liver recurrence who underwent systemic chemotherapy alone had poor outcomes, with a survival time of 28.7 months. This group of patients could not receive operation for various reasons, e.g., too advanced and poor general condition. Otherwise, randomized study is the only method to clarify which is better for remnant liver metastases, chemotherapy, operation, or combination chemotherapy with repeated operation. But such a study is also prohibitive due to ethical concerns. The 5-year survival rate in multi hepatectomy cases and in chemotherapy cases are 40~50% and 24.2%, respectively. This rate shows operation with systemic chemotherapy achieves cure as well as single hepatectomy cases for the cases with poor prognostic factors of repeat metastases. Thus, combining repeat hepatectomy with systemic chemotherapy is considered beneficial. In previous reports, Jones et al. [3] stated that repeat hepatectomy was not sufficient for prolonging survival time, whereas Battula et al., Freire, and Andreou et al. reported the effectiveness of aggressive repeat hepatectomy for prolonging survival time [6–9]. Recent reports have documented the survival-prolonging effects of repeat hepatectomy; thus, it is speculated that perioperative and backup drugs have a survival-prolonging effect. On the other hand, Lee et al. reported that repeat hepatectomy for multiple recurrent foci does not contribute to prognosis. Therefore, indications for repeat hepatectomy warrant further discussion [10]. There was one (1.7%) death due to complications in a patient who underwent two hepatectomies. Although this finding was consistent with previous reports by various investigators [11], death occurred in two (15.3%) patients who underwent ≥ 3 hepatectomies, which signifies a rather high percentage, and it is possible that surgical stress was too high or underlying liver status was poor in these two patients. Therefore, when performing a second or subsequent hepatectomy, it is critical to carefully assess the functional reserve of the remnant liver. Other complications included infection, bile leak, embolism, and pleural effusion/ascites; however, hospital stay was not increased, indicating that the surgery was safe and feasible. Advances in systemic chemotherapy prolonged survival ≥ 30 months in patients with unresectable advanced/recurrent CRC. In addition, some studies have reported conversion of unresectable metastasis cases to resectable status due to the advent of molecular-targeted drugs with a survival-prolonging effect, and such conversion cases have prolonged survival [12–15]. However, these reports provided only limited data on repeat resection following remnant liver recurrence. In the present study, we administered 2–5 systemic chemotherapy regimens to 55 patients, and 93% of patients undergoing repeat hepatectomy underwent multiple chemotherapy regimens. We believe in aggressively planning repeat hepatectomy when systemic chemotherapy has caused conversion to resectable status. Though the significance of preoperative chemotherapy in patients with resectable foci at the time of recurrence remains unclear [16–18], hepatectomy should be performed whenever possible, and multimodality treatment, including chemotherapy, should be performed in such patients.

## Conclusion

Repeat hepatectomy with chemotherapy for mCRC is feasible and might achieve long-term survival in carefully selected patients.

## Abbreviation

CRC: Colorectal cancer
